# Integrated Transcriptomic and Epigenomic Analysis Reveals Mechanisms Underlying Melanotic Spot Formation in Red Tilapia (*Oreochromis* spp.)

**DOI:** 10.3390/ijms26094370

**Published:** 2025-05-04

**Authors:** Zhangru Qi, Jiaxiang Liu, Jiale Shi, Miaomiao Yin, Jialong Liu, Jiaxuan Fan, Zhenmin Bao, Zhi Ye, Jingjie Hu

**Affiliations:** 1 Key Laboratory of Tropical Aquatic Germplasm of Hainan Province, Sanya Oceanographic Institution, Ocean University of China, Sanya 572024, China; qizr2001@163.com (Z.Q.); weifang1999ljx@163.com (J.L.); saltyle@163.com (J.S.); mm990117@163.com (M.Y.); liujialong@stu.ouc.edu.cn (J.L.); fanjiaxuan0415@163.com (J.F.); zmbao@ouc.edu.cn (Z.B.); 2 MOE Key Laboratory of Marine Genetics and Breeding, College of Marine Life Sciences, Ocean University of China, Qingdao 266003, China

**Keywords:** epigenetic regulation, histone modification, skin coloration, CUT&Tag, cis-regulatory elements

## Abstract

Red tilapia is highly valued as a premium variety in Asia due to its vibrant red skin coloration. However, during aquaculture production, irregular black pigmentation (melanotic spots) frequently appears on the skin of some individuals, significantly reducing their economic value. Although epigenetic regulation is suspected to play a role, its involvement remains poorly understood. To uncover the molecular mechanisms underlying black spot formation, we employed Cleavage Under Targets and Tagmentation (CUT&Tag) to compare four key histone modifications (H3K4me3, H3K4me1, H3K27me3, and H3K27ac) between red and black pigmented skin regions. Integrated with transcriptomic analysis, our data indicated that red skin regions exhibited high expression of genes suppressing melanin synthesis, whereas melanotic spots likely resulted from localized derepression, allowing upregulation of melanin biosynthetic genes. Furthermore, by combining epigenomic chromatin state analysis and transcriptome data, we identified critical genes consistently active in melanotic spots and their corresponding potential cis-regulatory elements. Motif analysis of transcription factor binding sites upstream of these regulatory elements revealed that Ehf, Klf9, and Egr1 might facilitate melanin production in black regions, while Prdm1 and Sp5 could inhibit melanogenesis in red regions by repressing the Wnt signaling pathway. These findings provide valuable epigenetic insights into the mechanisms driving melanotic spot formation in red tilapia.

## 1. Introduction

Red tilapia (*Oreochromis* spp.) is a hybrid strain obtained through artificial breeding screening, primarily by crossing the red-orange mutant of Mozambique tilapia (*Oreochromis mossambicus*) with species such as Nile tilapia (*Oreochromis niloticus*) or blue tilapia (*Oreochromis aureus*) [1]. This species not only has strong disease resistance and good adaptability, but also is popular among consumers because of its tasty meat, bright color and absence of black peritoneum. Thus, its economic value is much higher than common tilapia, and has gradually become an important economic species in aquaculture in several regions [2]. However, during the breeding process, red tilapia often exhibit trait segregation, and some individuals show irregular pigmentation, such as black spots on the skin, which affects their market appeal and reduces their economic value. Therefore, controlling the stability of body color and avoid the appearance of black spots through genetic improvement has become an important challenge in the current breeding and commercial culture of red tilapia, and solving this challenge relies on an in-depth understanding of the molecular mechanism of black spot formation.

The body color of fish is one of its important phenotypic features, determined by the specific composition and distribution pattern of pigment cells [3]. Four types of pigment cells have been identified in red tilapia, namely melanocyte, xanthophore, erythrophore, and iridocyte [4]. The color-presenting substance of melanocytes is melanin, and melanin synthesis involves numerous regulatory factors and signaling pathways, a process that is highly conserved in vertebrates [5]. Xanthophore and erythrophore both have pteridine and carotenoids as their color presenting substances, but they differ in their content [6].

Due to trait segregation, red tilapia may differentiate into all red, red with black spots, all pink, or pink with black spots [4]. It has been found that melanin synthesis is mainly regulated by genes such as *tyr* and *tyrp1*, which show different expression levels in red tilapia of different skin color types [7]. The transportation and storage of carotenoids are closely related to lipid metabolism, and genes such as *scarb1*, *plin2*, and *star* play key roles in the accumulation of carotenoids in red tilapia [8,9,10]. In addition, miRNAs such as miR-138-5p and miR-722 regulate the expression of genes related to melanin synthesis by targeting genes such as *mc1r* and *tyrp1*, thus regulating the formation of black spots in red tilapia [11]. Recent studies have shown that alternative splicing (AS) also plays a key role in skin color variation in red tilapia, further enriching the molecular mechanism of skin color variation in red tilapia [1]. The skin color of red tilapia is not only influenced by genetic factors, but also regulated by environmental factors. Temperature changes have a significant effect on skin pigment synthesis in red tilapia, and during overwintering, irregular black spots appear on the skin of some individuals, a process that may be related to changes in skin melanocytes and tyrosinase activity [12]. In addition, the culture background color also has a significant effect on the skin color of red tilapia, and the fish will regulate the distribution of pigment cells according to the background color, showing different skin colors [13].

From the above studies, we can hypothesize that the appearance of black spots in individual red tilapia is due to altered gene expression patterns in some cells in the skin. Although all cells of the same red tilapia have the same gene composition, they show differences in the expression levels of body color-related genes, resulting in the appearance of irregular black spots, and this non-programmed differential expression of genes is usually associated with epigenetic regulation. Histone modification is an important component of epigenetic regulation, playing a crucial role in gene expression regulation by affecting the open state of chromatin [14]. However, there have been no reports on the relationship between histone modification and the formation of black spots in red tilapia. Histone modification, as a key epigenetic regulatory mechanism, encompasses acetylation, methylation, ubiquitination, phosphorylation, and other forms, and often occurs in gene expression regulatory regions, which in turn affects gene expression by altering the structure and accessibility of chromatin [15]. For example, H3K4me1 is usually found in enhancer regions and is associated with gene activation [16,17,18]. H3K4me3 is usually found in transcription start sites (TSS) and is associated with active transcription of genes [19,20,21,22]. H3K27ac is the major epigenetic mark of active chromatin, and by altering the state of charge of histone proteins, it results in a more relaxed chromatin, thereby improving DNA accessibility and facilitates the binding of transcription factors [23,24,25]. H3K27me3 is often associated with Polycomb complexes, which are usually associated with gene silencing or repression [26,27,28]. Some studies have shown that epigenetic regulation, such as histone modification, is closely related to body color formation in animals. Histone Deacetylase 1 (Hdac1) affects melanocyte development and melanogenesis in zebrafish (*Danio rerio*) by repressing the *foxd3* gene and indirectly promoting *mitfa* expression [29,30]. Histone acetyltransferase p300/CBP enhances the acetylation of H3K27 histone, amplifying the expression of differentiation genes downstream of microphthalmia-associated transcription factor (MITF), thereby enhancing melanocyte differentiation [31]. In addition, Tan et al. revealed an epigenetic signature between two different shell colors by comparing histone methylation levels in individual pacific oysters (*Crassostrea gigas*) [32].

This study aims to investigate the gene expression and epigenetic regulatory mechanisms of black spot formation in red tilapia skin. Using RNA-Seq technology, we analyzed the gene expression differences between the black spots and the surrounding red regions in red tilapia skin tissues, identified the expression patterns of related genes, and screened key genes affecting black spot formation. Meanwhile, using CUT&Tag technology, we conducted a comparative analysis of the genome-wide modification status of four important histone modifications (H3K4me1, H3K4me3, H3K27ac, and H3K27me3) in the skin tissues of the black spots region and its surrounding red area, to identify histone modification regulatory sites related to the formation of black spots. Finally, the epigenomic chromatin state was used to analyze the joint transcriptome for significant motif sites, which led to the prediction of key transcription factors and revealed a plausible regulatory mechanism of black spots formation in red tilapia. Moreover, this study will provide a theoretical basis for revealing the molecular mechanism of black spots formation in red tilapia and provide a scientific basis for red tilapia species improvement and culture optimization.

## 2. Results

### 2.1. Transcriptome Data Processing and Alignment

Total RNA was successfully extracted from skin samples collected from both the black spot regions and the surrounding red areas, and all samples met the quality requirements for RNA library construction. Sequencing yielded an average of 26,409,603 pairs of raw reads per sample. After quality filtering, an average of 26,181,701 pairs of clean reads were obtained, with an average Q30 score of 94.78% (Table S1). Nile tilapia is one of the parents of red tilapia, so we chose this genome as the reference genome (RefSeq assembly accession: GCF_001858045.2). The percentage of clean reads matching to the reference genome for each library was more than 94%, and the percentage of reads uniquely matching to the reference genome was higher than 89% (Table S2). This indicates that the sequencing data are of good quality and the reference genomes are well adapted for subsequent analysis.

### 2.2. Transcriptome Differential Expression and Enrichment Analysis

**Figure 1:**
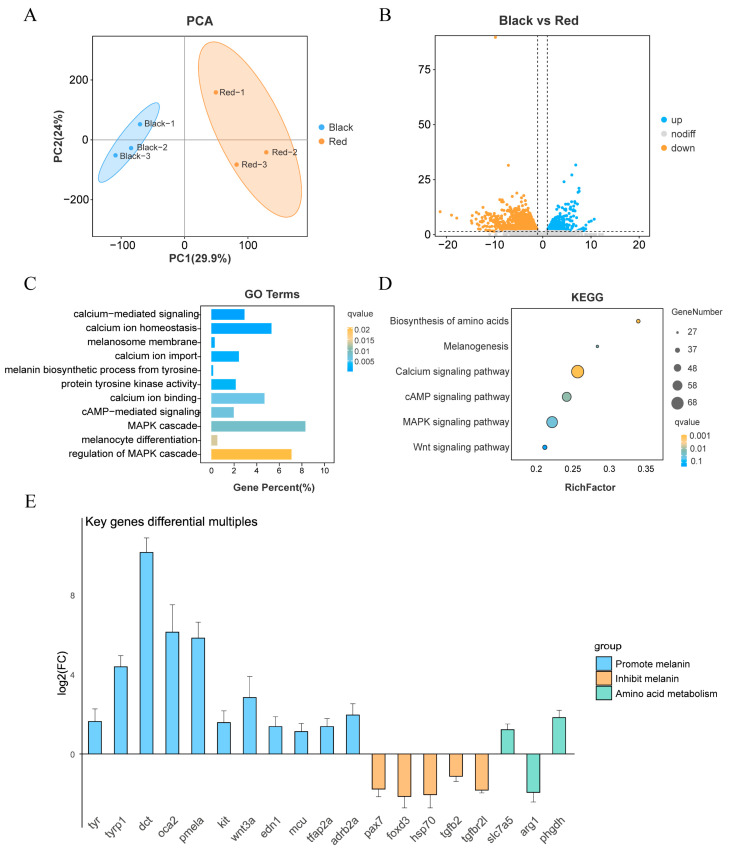
Transcriptome analysis of Red Tilapia (**A**) PCA plot of RNA-Seq samples from the black spot region and red region. Black: black spot region; Red: red region. Numbers 1, 2, and 3 represent biological replicates. (**B**) Volcano plot of DEGs between the black spot region and red region. *p* < 0.05 and |log_2_FC| > 1 were considered significant DEGs. The −log10 of the *p*-value is used for the Y-axis, and the log_2_ of the fold change (log_2_FC) is used for the X-axis. Blue dots represent up-regulated genes, yellow dots represent down-regulated genes, and gray dots represent genes with no significant differences. (**C**) GO enrichment analysis of DEGs with terms potentially related to black spot formation. The Y-axis represents GO terms, the X-axis represents the proportion of genes, and the color of the bars represents the *q*-value. (**D**) Kyoto Encyclopedia of Genes and Genomes (KEGGs) enrichment analysis of DEGs with pathways potentially related to black spot formation. The Y-axis represents KEGG pathway names, and the X-axis represents RichFactor; the size of the points represents the number of genes, and the color represents the *q*-value. (**E**) Fold changes in the expression of key genes. Blue represents genes that promote melanin synthesis, orange represents genes that inhibit melanin synthesis, and green represents genes that affect amino acid metabolism. The Y-axis represents log_2_FC, and the X-axis represents gene names. Genes above the X-axis are upregulated in the black spot region, and genes below the X-axis are downregulated in the black spot region.

The principal component analysis (PCA) of the transcriptome data of the black spots region and the red region is shown in Figure 1A. From the figure, it can be seen that there are significant differences between these two groups. Differential expression analysis of the transcriptome sequencing results yielded a total of 4862 differentially expressed genes (DEGs), of which 1841 genes were up-regulated and 3021 genes were down-regulated in the black spots region (Figure 1B). Gene ontology (GO) enrichment analysis yielded a total of 1089 significantly enriched GO entries (*q* < 0.05). Among them that may be related to melanin synthesis include calcium-mediated signaling, melanin biosynthetic process from tyrosine, melanosome membrane, regulation of MAPK cascade, cAMP-mediated signaling (Figure 1C).

A total of 344 pathways were obtained from the KEGG pathway enrichment analysis, of which 32 pathways were significantly enriched (*q* < 0.05). KEGG pathway enrichment yielded a total of 344 pathways, including 32 significant pathways (*q* < 0.05). These include calcium signaling pathway, biosynthesis of amino acids, cAMP signaling pathway, melanogenesis, MAPK signaling pathway, etc. In addition, Wnt signaling pathway was also enriched (*p* < 0.05). Combined with previous studies [33], the pigmentation of the skin in the black spots region may be closely related to the activities of these pathways (Figure 1D).

GO enrichment and KEGG enrichment were used to further narrow down the genes that may affect black spots formation in red tilapia. We found that genes promoting melanin synthesis such as *tyr*, *tyrp1*, *dct*, *oca2*, *LOC100711455 (pmela)*, *kit*, *wnt3a*, *edn1*, *mcu*, *tfap2a*, and *LOC100706475 (adrb2a)* were significantly up-regulated in black spots. Interestingly, we found the expression of a series of genes that inhibit melanin synthesis in the red region, including *pax7*, *foxd3*, *LOC100534448 (hsp70)*, *tgfb2*, and *LOC100692823 (tgfbr2l)*, suggesting that red tilapia may need to maintain red coloration of the body by inhibiting melanin synthesis by default, and that the emergence of black spots region may be the result of this inhibition being lifted as a result. In addition, we found that genes related to amino acid metabolism, such as *slc7a5*, *LOC100706482 (arg1)*, and *phgdh*, differed significantly between the black spots region and the red region, and may regulate melanogenesis by affecting tyrosine supply. The fold changes in the expression of the aforementioned genes are shown in Figure 1E.

### 2.3. CUT&Tag Data Processing and Alignment

A total of 20 sets of data were obtained by sequencing, including different histone modification marks in black spots site and red site, with two replicates for each histone modification, and an average of 6,115,737 pairs of raw reads were obtained. 5,140,561 pairs of clean reads were obtained on average after filtering, and the Q30s were all higher than 91.59%. The sequencing data were of high quality and suitable for subsequent bioinformatics analysis, as detailed in Table S3.

Genome matching was performed after quality control of the sequencing data, and most samples showed a matching rate of more than 81%. The Spike-in matching rate was below 2.5% for most samples, with only the B_IgG and R_IgG groups showing higher rates of 10.59% and 8.42%, respectively, which is in line with the expectation of obtaining less specific fragments for the IgG control group, suggesting that the Spike-in matching rate is within a reasonable range (Table S4). These results indicate that the quality of the obtained CUT&Tag data is reliable and suitable for subsequent downstream analysis.

### 2.4. Distribution and Enrichment Characteristics of Histone Modification Signals in the Skin Tissue of Black Spot and Red Regions

**Figure 2:**
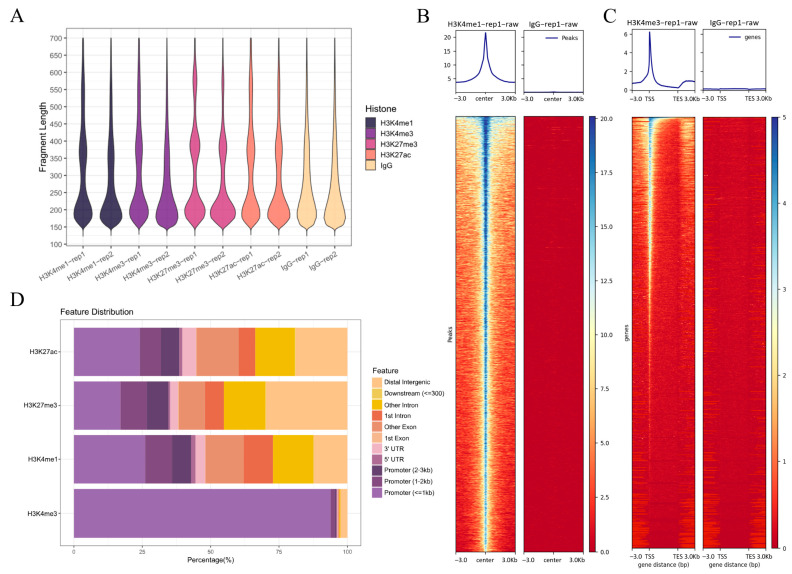
Distribution and enrichment features of histone modification signals (**A**) Fragment length distribution of CUT&Tag samples in the black spots region. (**B**) Heatmap of the H3K4me1 peak region in the black spots region. (**C**) Heatmap of the H3K4me3 TSS site region in the black spots region. (**D**) Distribution of Peak in the functional region in the black spots region. rep: biological replicates.

Analysis of replicate samples of skin tissue from the black spots region showed that the size of the sheared DNA fragments was mainly clustered at approximately 200 bp and 350 bp (Figure 2A), with similar red regions (Figure S1). This result is consistent with the expectation of a gradient distribution of nucleosome DNA fragment sizes, demonstrating that the experiments were able to efficiently capture target histone-associated DNA fragments. Statistical analysis of H3K4me1, H3K4me3, H3K27ac, and H3K27me3 histone modification signals in two sets of replicate samples yielded the average number of specific modification signals detailed in the skin tissue (Table 1).

Compared to the negative control IgG, four histone modifications (H3K4me1, H3K4me3, H3K27ac, and H3K27me3) exhibited significant signal enrichment in both the black spot and red regions, with H3K4me1 in the black spot region as an example (Figure 2B). In addition, in the black spot region, H3K4me3 showed strong enrichment at the TSS (Figure 2C), which is consistent with the distribution preference of histone modification signals. The remaining histone modification signaling heatmaps are shown in Figure S2. From the perspective of genomic distribution, H3K4me3 signals were mainly concentrated in the promoter region (particularly within the 1–2 kb and ≤1 kb ranges), while H3K27ac, H3K4me1, and H3K27me3 signals were mainly distributed in exons, introns, and distal intergenic regions, with only a small portion located in the promoter region (Figure 2D). The results for the red region were similar (Figure S3). This distribution reflects the differential enrichment of various histone modifications across genomic features, which is closely related to their distinct biological functions in gene regulation.

### 2.5. Comparison and Analysis of Differential Histone Modification Signals

Differential analysis of histone modification signals in the black spot and red region samples was performed. The modifications H3K4me1, H3K4me3, H3K27ac, and H3K27me3 corresponded to 3542, 564, 1175, and 2274 differential peaks, respectively. It is important to note that the same gene may have multiple differential peaks in different regions. These differential peaks do not directly represent the number of DEGs, as a gene may exhibit histone modification changes at multiple locations. Therefore, to accurately identify differential genes, we annotated these differential peaks to ensure that each peak was correctly associated with the corresponding gene, thus identifying the differential genes at the gene level. After annotation, we obtained 2765, 562, 1032, and 1952 differential genes, respectively (Table 2).

**Figure 3:**
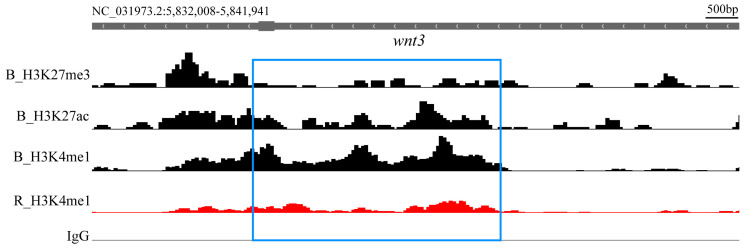
Shows the comparison of H3K4me1 histone modification signal intensities for the *wnt3* gene between the black spot and red regions in the Integrative Genomics Viewer (IGV), with the differential sites identified in the differential analysis highlighted within the blue box. The H3K27ac and H3K27me3 histone modification signals in the black spot region are also displayed, indicating that this site is an active enhancer in the black spot region. B: Black spot region; R: Red region.

Upon analyzing these differential genes, we found that several genes promoting melanin synthesis exhibited differential H3K4me1 signals between the black spot and red regions, including *ctnnb1*, *wnt3*, *LOC112846512 (camk2d)*, *creb3l2*, *kitlg*, *LOC102082978 (edn3)*, and *bambi*. Additionally, the *kit* and *kitlg* genes showed differential H3K27ac signals. Furthermore, several signal sites inhibiting melanin production in the red region showed differences, including *nf1*, *LOC100696396 (foxo6b)*, *LOC100698110 (dkk1)*, and genes that play an important role in the TGF-β signaling pathway, such as *LOC106098551 (tgfbr2l)* and *LOC100700558 (smad4).* The differential information for the aforementioned gene sites is shown in Table 3. As an example, the comparison of histone modification signal intensities between the black spot and red regions for *wnt3* is shown in Figure 3.

### 2.6. Chromatin State Analysis of the Black Spot and Red Regions

ChromHMM uses multiple histone modification marks to systematically annotate the genome. It divides the genome into several small intervals using a multivariate hidden Markov model, determines the presence of each mark, and infers different chromatin states. These states represent different functional regions in the genome, such as promoters, enhancers, transcriptionally active regions, and repressive regions [47].

**Figure 4:**
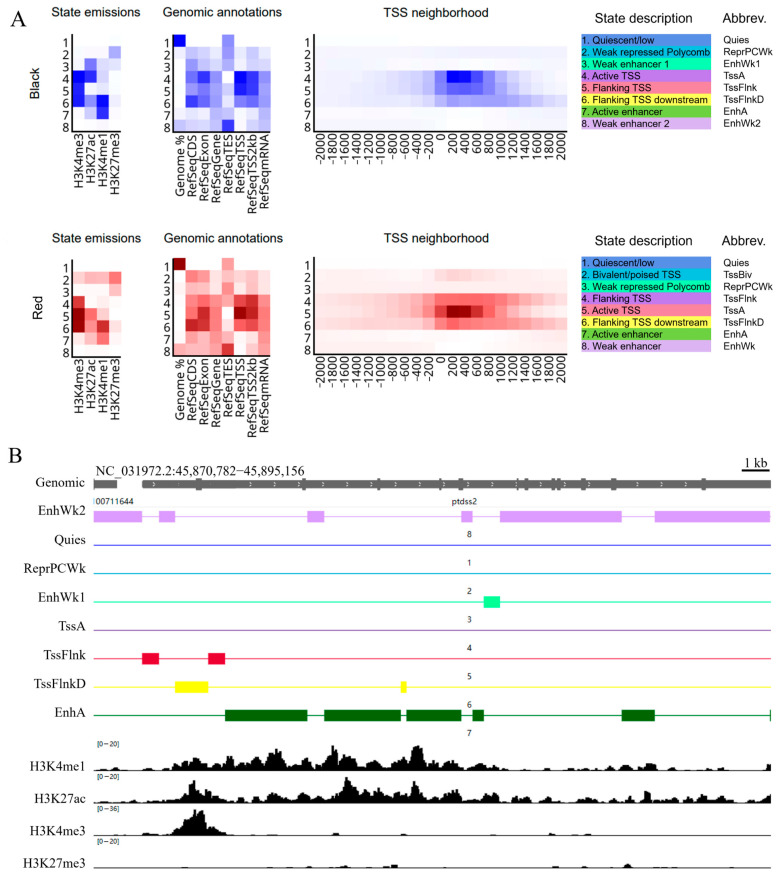
Chromatin State Analysis (**A**) Distribution of chromatin states in the genome for the black spot and red regions. State emissions show the distribution of different histone modifications across chromatin states, with the intensity of the color reflecting the strength of these modification signals. Genomic annotations provide genomic annotation information, including exons, genes, and other features. TSS neighborhood shows the distribution of chromatin states near the TSS. The legend State description represents various chromatin states, with colors corresponding to each state in IGV. Abbrev represents abbreviations of chromatin state names. (**B**) Comparison of the chromatin state analysis results for the *ptdss2* gene in the black spot region with the CUT&Tag analysis results for individual histone modifications. The left side shows the abbreviations of chromatin state names and histone modification names.

Through ChromHMM computation and annotation, the distribution of chromatin states in the genome for the black spot and red regions was obtained (Figure 4A). State emissions show the distribution of different histone modifications (H3K4me1, H3K4me3, H3K27ac, and H3K27me3) across chromatin states. The intensity of the color reflects the strength of these modification signals, helping to distinguish different chromatin regions, such as enhancers and promoters. Genomic annotations provide genomic annotation information, covering exons, genes, transcription termination sites (TES), TSS, and the 2 kb region around the TSS, which can be used to locate the distribution of chromatin states in the genome. TSS neighborhood shows the distribution of chromatin states near the TSS, particularly the signals in the active TSS region. The legend state description represents various chromatin states, such as weak enhancer, active enhancer, active transcription start site, and bivalent transcription start site, further revealing their potential roles in gene regulation. As an example, in the black spot region, a randomly selected gene was analyzed, and the results of the chromatin state analysis were compared with the CUT&Tag analysis results, as shown in Figure 4B. The results obtained from both methods are consistent, validating the reliability of the ChromHMM analysis results.

### 2.7. Chromatin State and Transcriptome Integrated Analysis

We observed consistent overall trends in the results of the transcriptome and histone modification differential analyses. For example, genes promoting melanin synthesis in the black spots region and genes inhibiting melanin synthesis in the red region showed differences in both transcriptomic and epigenomic analyses. However, since the RNA-Seq and CUT&Tag experiments involved in this study were performed based on whole skin tissue samples, cellular heterogeneity may mask some signals. In addition, histone modification is only one aspect of gene expression regulation, mRNA levels are also affected by post-transcriptional regulation and the stability of transcripts varies across genes [48]. These factors make it challenging to analyze the association between histone modifications and RNA-Seq data. We infer that the formation of black spots in red tilapia is the result of the long-term accumulation of differential expression in key genes, and these genes maintain differential histone modification states in both the black spot and red regions, leading to downstream gene expression differences. Therefore, we jointly analyzed the gene expression levels with the consistency of chromatin opening status.

**Figure 5:**
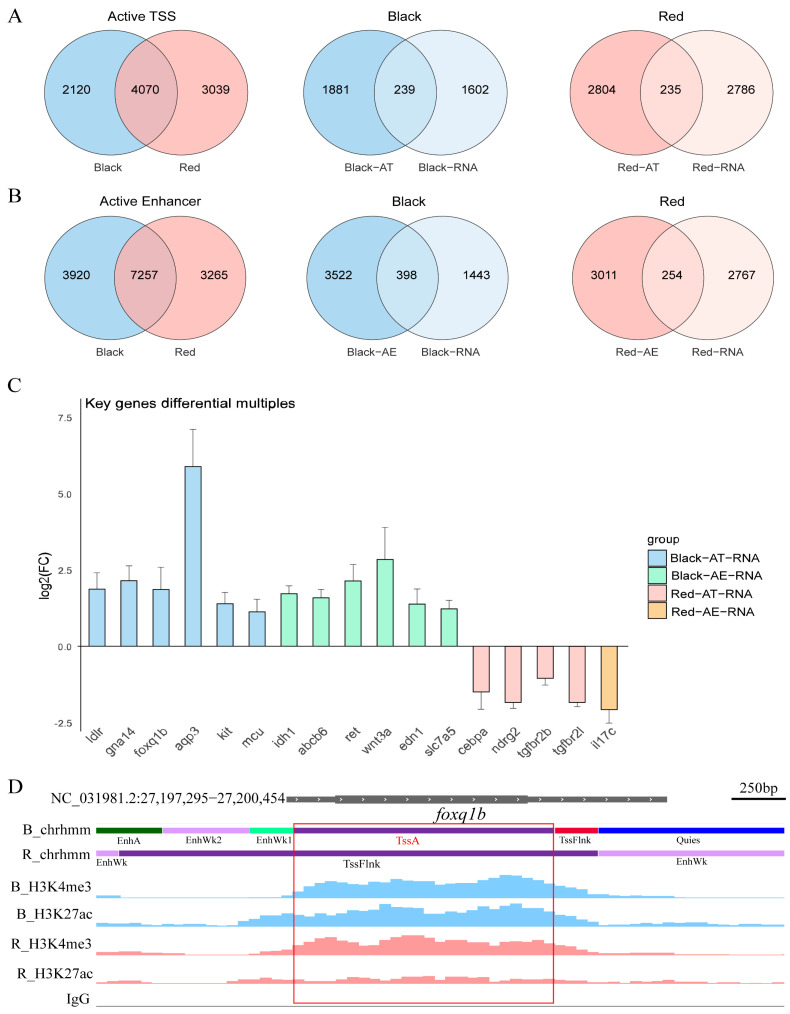
Chromatin state and transcriptome analysis of the red tilapia. (**A**) Active TSS sites unique to the black spot and red regions: The active TSS sites specific to the black spot region were intersected with the upregulated genes in the transcriptome of the black spot region, resulting in Black-AT-RNA. The active TSS sites specific to the red region were intersected with the upregulated genes in the transcriptome of the red region, resulting in Red-AT-RNA. (**B**) Active enhancer sites unique to the black spot and red regions: The active enhancer sites specific to the black spot region were intersected with the upregulated genes in the transcriptome of the black spot region, resulting in Black-AE-RNA. The active enhancer sites specific to the red region were intersected with the upregulated genes in the transcriptome of the red region, resulting in Red-AE-RNA. (**C**) Differential expression of key intersecting genes: Different colors represent different groups. The Y-axis represents log_2_FC, and the X-axis represents the gene names. Genes with expression levels upregulated in the black spot region are shown above the X-axis, while genes with expression levels downregulated in the black spot region are shown below the X-axis. (**D**) Differential H3K4me3 and H3K27ac histone modifications for the *LOC100698267 (foxq1b)* gene. The red box highlights the comparison of signal intensity for the differentially identified sites. B: Black spot region; R: Red region; chrhmm: Chromatin state analysis.

Active TSSs are genomic regions near the transcription initiation sites that are in an active transcriptional state, reflecting ongoing gene expression. By comparing the active TSSs identified in the black spot regions with those in the red skin regions, and extracting the genes uniquely associated with each, we identified 2120 genes specific to the black spot regions and 3039 genes specific to the red skin regions. To further investigate the correlation between chromatin activity and gene expression, we intersected region-specific active TSSs with the upregulated genes identified by transcriptome analysis. This yielded 239 genes in the black spot region (Black-AT-RNA) and 235 genes in the red region (Red-AT-RNA) (Figure 5A). Similarly, active enhancers, which are defined as genomic enhancer regions exhibiting transcriptional activity, were analyzed using the same approach. The resulting gene sets were designated as Black-AE-RNA and Red-AE-RNA, respectively (Figure 5B).

The unique active TSS sites in the black spot region were intersected with the upregulated genes in the transcriptome of the black spot region, resulting in multiple genes related to melanin synthesis, including *ldlr*, *LOC100706143 (gna14)*, *LOC100698267 (foxq1b)*, *LOC100708473 (aqp3)*, *LOC100711613 (kit)*, and *mcu*. The unique active enhancer sites in the black spot region were intersected with the upregulated genes in the transcriptome of the black spot region, also identifying several genes related to melanin synthesis, including idh1, *LOC100696961 (abcb6)*, *ret*, *wnt3a*, *edn1*, and *slc7a5*. The unique active TSS sites in the red region were intersected with the upregulated genes in the transcriptome of the red region, revealing several genes that inhibit melanin synthesis, including *cebpa*, *LOC100708554 (ndrg2)*, *LOC100712573 (tgfbr2b)*, and *LOC100692823 (tgfbr2l)*. The unique active enhancer sites in the red region were intersected with the upregulated genes in the transcriptome of the red region, identifying the *LOC100708542 (il17c)* gene, which may influence melanin production. The differential expression of these genes in the transcriptome is shown in Figure 5C.

It is generally accepted that sites with both H3K4me3 and H3K27ac modifications are active transcriptional start sites. In order to verify the accuracy of the chromatin state analysis of ChromHMM, we took the *LOC100698267 (foxq1b)* gene as an example, and the differences in its histone modifications are shown in Figure 5D. In the figure, the red box shows the region identified by ChromHMM as the black spots-specific active TSS element, it can be seen that there is no significant difference in the H3K4me3 modification of this segment of the gene, but the H3K27ac modification is significantly different in the black spots region and the red region, so this element is determined as active TSS in the black spots region and flanking TSS in the red region. Thus, it can be seen that the chromatin state analysis of ChromHMM is accurate and can identify the gene expression regulatory elements of these key genes. Additional manual inspections of other genes revealed similar patterns, further validating the accuracy of our method for identifying region-specific active TSS elements.

### 2.8. Motif and Transcription Factor Prediction

**Figure 6:**
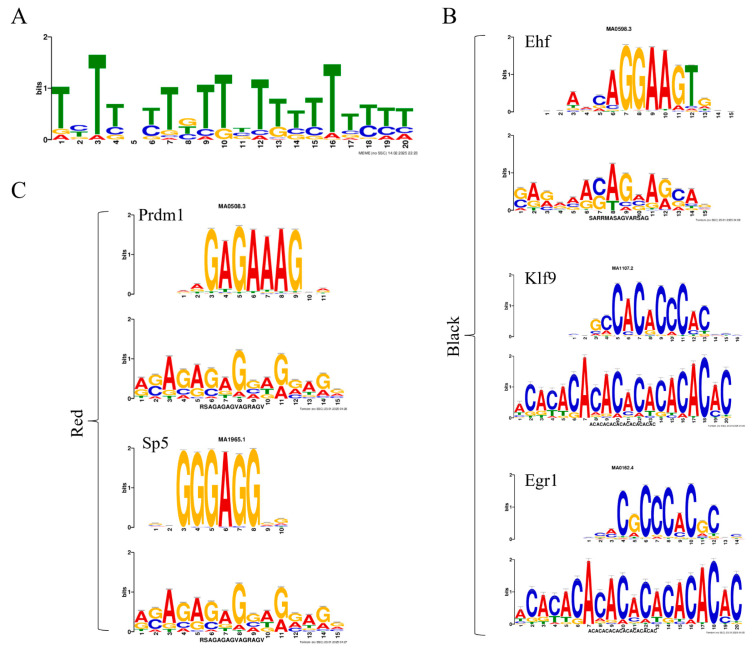
Motif and Transcription Factor Prediction. (**A**) A significant motif site in Black-AT-RNA. The Y-axis reflects the conservation of the position, with higher values indicating greater conservation of nucleotides at that position across different sequences. The X-axis shows the positions of nucleotides in the motif sequence. The same applies below. (**B**) Three important transcription factors (Ehf, Klf9, Egr1) predicted in the black spot region. Taking Ehf as an example, the first row represents the motif recognized by the transcription factor, and the second row shows the motif sites we identified. The same applies below. (**C**) Two important transcription factors (Prdm1, Sp5) predicted in the red region.

All intersecting genes obtained from each set were subjected to motif prediction separately. Taking one of the motifs of Black-AT-RNA as an example, the schematic diagram is shown in Figure 6A, and the rest are shown in Figure S4, after which the transcription factors corresponding to each motif were predicted separately. Black-AT-RNA as well as Black-AE-RNA were predicted to obtain the transcription factor ETS homologous factor (Ehf), which was significantly up-regulated in the black spots region expression was significantly upregulated (*p* = 0.0066, log_2_(fc) = 2.09), speculating that Ehf may have an important regulatory role in black spots formation. It is very interesting to note that binding sites for this transcription factor are present on the key genes identified in Section 2.7 that promote melanin synthesis (excluding *idh*). In addition, the transcription factors Kruppel-like factor 9 (Klf9) and early growth response 1 (Egr1), etc. were also predicted. Black-AT-RNA is an example, and the sequence information of the three transcription factors mentioned above is shown in Figure 6B.

Both Red-AT-RNA and Red-AE-RNA predict the transcription factors PR domain zinc finger protein 1 (Prdm1) and specificity protein 5 (Sp5), and both show a significant upregulation of expression in the red region. *Prdm1* (*p* = 2.53 × 10^−6^, log_2_(fc) = −2.4709) and *sp5* (*p* = 0.0005, log_2_(fc) = −5.3837). Therefore, we can speculate that these two factors have an important impact on melanogenesis. Furthermore, we found that this transcription factor may interact with the key gene in suppressing melanogenesis in Section 2.7 (except for *il17c*). Taking Red-AT-RNA as an example, the sequence information of the two transcription factors is shown in Figure 6C.

## 3. Discussion

### 3.1. Gene Regulatory Network and the Molecular Mechanism of Spot Formation

In this study, transcriptome data analysis identified gene expression differences between the black spot region and the red region in red tilapia. In the black spot region, key genes involved in melanogenesis (such as *tyr*, *tyrp1*, and *dct*) were significantly upregulated. Additionally, the *oca2* and *pmela* were highly expressed in the black spot region. These genes regulate the pH of the melanosomal environment and maintain the levels of tyrosinase protein, thereby influencing the function of tyrosinase [49,50]. The high expression of these genes is associated with the stable synthesis of melanins in the black spot region, which is consistent with the findings of Huang Junrou et al. [1,4]. We also found that the *kit*, *wnt3a*, *edn1*, and *mcu* were significantly upregulated in the black spot region. The *kit* encodes the KIT proto-oncogene, receptor tyrosine kinase, c-KIT, which, upon binding with the ligand stem cell factor (SCF), can activate the downstream MAPK signaling pathway and upregulate the transcriptional activity of MITF [41]. The *wnt3a* encodes a key regulatory factor in the Wnt signaling pathway, which induces *mitf* expression and activates the MITF promoter by recruiting lymphoid enhancer-binding factor 1 (LEF-1) and β-catenin to LEF-1 binding sites [35]. The *edn1* encodes endothelin-1 (ET-1), which, upon binding to endothelin receptor type B (EDNRB) in an autocrine manner, activates the cAMP signaling pathway to participate in pigmentation [51]. The *mcu* encodes the mitochondrial calcium uniporter (Mcu), and the enhancement of mitochondrial calcium signaling is directly related to an increase in melanogenesis. It has been demonstrated in mice (*Mus musculus*) and zebrafish that Mcu enhances the melanogenesis process by affecting the expression of key enzymes involved in melanogenesis, such as Tyr, Tyrp1, and Dct [52]. Additionally, the synergistic effect of *tfap2a* and *mitf* may form a core regulatory module for melanocyte differentiation. Their dual mutation leads to a more pronounced loss of pigment cells, suggesting that these two factors may have functional conservation in evolution [53]. Moreover, in zebrafish, it has been found that *adr2b* may further enhance melanogenesis by antagonizing the negative regulatory effect of F1F0-ATPase [54]. These multi-level regulatory mechanisms collectively explain why the black spot region can maintain a continuous ability for pigment deposition.

In the red region, an inhibitory regulatory network limits melanogenesis. In neural crest cells, the paired box 7 (Pax7) encoded by the *pax7* gene, can inhibit Mitf to drive the transcription of the xanthophore marker gene *gch2* [5]. In mature melanocytes, overexpression of *foxd3* blocks the expression of *mitf*, thereby inhibiting the expression of genes related to melanogenesis [55]. Additionally, *hsp70* encodes heat shock protein 70, which affects the activity of MITF by directly interacting with it, thereby inhibiting the transcription and activity of tyrosinase [56]. The TGF-β pathway inhibits the transcriptional regulator paired box 3 (Pax3), thereby suppressing MITF expression and reducing melanin synthesis [45]. The *tgfb2* and *tgfbr2l* genes are highly expressed in the red region and can inhibit melanogenesis by activating the TGF-β signaling pathway [57]. Combined with the promoting effect of Pax7 on xanthophore differentiation, we speculate that the pigment pattern in the red region of red tilapia is shaped by two mechanisms: “inhibiting melanogenesis” and “promoting the development of other pigment cells”. This bidirectional regulatory pattern may be similar to the competitive mechanism between melanocytes and xanthophore in zebrafish stripe formation [58], suggesting an evolutionary conservation of pigment pattern regulation in teleosts.

In addition, amino acid metabolism may also have an impact on melanogenesis. Studies have found that *slc7a5* regulates melanogenesis by controlling the supply of tyrosine, and inhibiting *slc7a5* significantly reduces melanin synthesis [59]. The *arg1* encodes arginase-1 (ARG1), and Eda et al. discovered that inhibition of ARG1 activity enhances melanogenesis through the p53 signaling pathway [60]. Therefore, *arg1* may play a negative regulatory role in melanogenesis, and this gene is significantly downregulated in the black spot region. Furthermore, we also found that the *phgdh* is significantly upregulated in the black spot region. In mice, it has been found that overexpression of *phgdh* increases the number of melanocytes in the skin, possibly by influencing one-carbon metabolism through serine synthesis, indirectly regulating melanogenesis [61].

In summary, transcriptome data reveal that the black spot region forms sustained melanin deposition ability by activating multiple signaling pathways and upregulating key melanogenesis genes, while the red region limits melanin synthesis through multiple inhibitory mechanisms. These findings lay the foundation for the next step in exploring the role of epigenetic regulation in regional pigment differentiation.

### 3.2. Histone Modification Screening of Key Regulatory Elements

Using CUT&Tag technology for histone modification analysis, we found multiple key gene loci promoting melanogenesis with differential H3K4me1 signals. The *ctnnb1* encodes β-catenin, which promotes melanin synthesis by activating MITF and its downstream genes [34]. Additionally, the H3K4me1 signal of the *wnt3* gene in the black spot region was significantly upregulated. Wnt3 promotes melanogenesis by upregulating MITF, TYR, and TRP1 [35]. Therefore, we hypothesize that the Wnt signaling pathway may drive region-specific melanogenesis through epigenetic modifications. Furthermore, *camk2d* encodes calcium/calmodulin-dependent protein kinase II δ subunit (CaMKII), which plays an important role in calcium ion signaling. Calcium signaling activates CaMKII and further activates the AKT pathway, promoting MITF expression and subsequently increasing the expression levels of melanogenesis-related enzymes [36]. The *creb3l2* encodes cAMP response element-binding protein 3-like 2 (Creb3l2), and research in Pacific oysters has shown that Creb3l2 may be activated through the cAMP/PKA signaling pathway, and synergize with other transcription factors regulating melanogenesis (such as MITF), further promoting the expression of melanogenesis-related genes [37]. The *kitlg* encodes stem cell factor, a key regulator of melanogenesis. KITLG binds to its receptor c-KIT, activating the downstream RAS/MAPK signaling pathway, which in turn activates MITF and promotes melanogenesis [38]. Studies have shown that Edn3 collaborates with the Kit signaling pathway to promote melanocyte survival, proliferation, and melanogenesis [39]. In addition, the *bambi* gene exhibits significant differential H3K4me1 signals between the black spot region and the red region, with an upregulation of this signal in the black spot region. BMP and activin membrane bound inhibitor (BAMBI) competitively binds to the TGF-β receptor complex, blocking TGF-β signaling, thereby relieving the inhibition of MITF expression and indirectly promoting melanogenesis [40]. In conclusion, the black spot region may regulate a MITF-centered network through H3K4me1-mediated enhancer elements, collaboratively promoting melanocyte development, proliferation, and melanogenesis. Therefore, these cis-regulatory elements should be a focus in future studies.

In the red region, epigenetic modifications primarily manifest as activation modifications of genes that inhibit melanogenesis. The H3K4me1 signal of the *nf1* gene is upregulated. NF1 inhibits melanogenesis through a dual mechanism: by using its GAP activity to convert Rat Sarcoma (RAS) from the active GTP-bound state to the inactive GDP-bound state, inhibiting the MAPK signaling pathway, and by regulating the balance of the cAMP/PKA signaling network to control MITF activity. This multitarget regulation may provide NF1 with flexible regulatory capabilities [42]. FoxO6b regulates antioxidant enzymes (MnSOD and Catalase) to reduce reactive oxygen species (ROS) levels, thereby inhibiting the activation of DNA-binding transcriptional regulator CREB and tyrosinase, suggesting a negative feedback mechanism between oxidative stress and melanogenesis [43]. The H3K27me3 signal of *dkk1* in the red region is downregulated, which may inhibit MITF through the Wnt signaling pathway [62]. The downregulation of the H3K27me3 signal of *smad4* enhances the inhibitory effect of TGF-β signaling on MITF [46], which is consistent with the high expression of *tgfb2* and *tgfbr2l* in the transcriptome, activating the TGF-β signaling pathway.

Through CUT&Tag experiments, we found differential H3K4me1 signals at several key gene loci that promote melanogenesis, and multiple differential signaling loci that may inhibit melanogenesis in the red region. These results are highly consistent with the RNA-Seq findings, which will aid in the next step of multi-omics integration analysis for this study.

### 3.3. Epigenetic and Transcriptional Regulatory Networks of Pigment Differences Between the Black Spot and Red Regions in Red Tilapia

This study integrates epigenomic and transcriptomic data to reveal the molecular differences in melanogenesis regulation between the black spot and red regions. In the black spot region, an intersection analysis of specific active TSS and enhancer sites with upregulated genes suggests that several genes related to melanogenesis may promote pigment production through epigenetic regulation. In contrast, the intersection of specific regulatory elements with the transcriptome in the red region shows a trend of inhibiting melanogenesis.

In the black spot region, *ldlr* may enhance melanogenesis by regulating cholesterol metabolism [63]. Additionally, the upregulation of *gna14* may promote melanogenesis by activating G protein-coupled receptor (Gpcr) signaling, which is consistent with the regulation of skin pigmentation by *gna14* in zebrafish models [64]. The *foxq1b* encodes Foxq1b, a member of the Forkhead Box family of transcription factors. Foxq1b responds to upstream cAMP and MAPK signaling pathways, binding to the MITF promoter to activate MITF transcription [65]. The proteins encoded by the *aqp3* and *idh1* have antioxidant functions, playing a significant role in melanocyte survival and melanogenesis [66,67]. The *abca6* encodes ATP-binding cassette sub-family B member 6, mitochondrial (Abcb6), a member of the ABC transporter family. In human melanocytes, ABCB6 regulates the protein expression of MITF and three downstream melanogenesis enzymes (TYR, TYRP1, and TYRP2) through the Wnt/β-catenin pathway, thus regulating melanogenesis [68]. The ret gene encodes a receptor tyrosine kinase that induces high levels of melanogenesis during melanocyte development in mice, which is associated with the upregulation of melanogenesis [69]. In addition, the combined analysis of the *kit*, *mcu*, *wnt3a*, *edn1*, and *slc7a5* also promotes melanogenesis, as detailed in Section 3.1.

In the red region, the core of the inhibitory regulatory network is the antagonism of MITF. The *cebpa* encodes the transcription factor CCAAT/enhancer-binding protein alpha (CEBPA). Studies have shown that CEBPA binds to the enhancer region of *mitf*, silencing it and thereby inhibiting melanogenesis [70]. The *ndrg2* encodes N-myc downstream regulated gene 2 (Ndrg2), which can block cAMP and β-catenin-mediated activation of the MITF promoter, resulting in low levels of melanogenesis [71]. Additionally, *il17c* encodes interleukin-17C (IL-17C), a cytokine belonging to the IL-17 family. Zhou Jun et al. demonstrated that IL-17 induces a cellular stress microenvironment in melanocytes, inhibiting melanogenesis in zebrafish, normal human epidermal melanocytes, and B16F10 cells. Therefore, the high expression of this gene may lead to the apoptosis of melanocytes [72]. The *tgfbr2b* and *tgfbr2l* genes inhibit melanogenesis through the TGF-β signaling pathway, as detailed in Section 3.1.

Both Black-AT-RNA and Black-AE-RNA predicted the transcription factor Ehf. This transcription factor is significantly upregulated in the black spot region (*p* = 0.0066, log_2_(fc) = 2.09), suggesting that Ehf may play an important regulatory role in the formation of the black spot. Ehf is an ETS homologous factor, belonging to the ETS family, and plays a crucial role in the development and function of the immune system, nervous system, and epithelial cells [73,74,75]. Another transcription factor in the ETS family, E26 transformation-specific-1 (ETS-1), has been shown to play an important role in the development of melanocytes and melanogenesis. In mice, ETS-1 promotes melanoblast differentiation and survival by activating the enhancer region of *sox10*. Additionally, ETS-1 can suppress *sox9* expression, which may help prevent improper development during melanocyte differentiation [76]. Since the Ehf transcription factor shares the same domain as ETS-1, we speculate that Ehf may play an important role in regulating the formation of black spots in red tilapia. We also found binding sites for this transcription factor on key melanogenesis-related genes such as *ldlr* and *gna14*, and we hypothesize that Ehf may induce melanogenesis by binding to these genes. The transcription factor Klf9 was also predicted in both Black-AT-RNA and Black-AE-RNA. Studies have shown that Klf9 regulates oxidative stress (ROS) by increasing ROS levels, which in turn promotes the full activation of ERK1/2, enhancing the proliferation capacity of melanocytes. ROS play an important role in melanogenesis because they may promote melanin synthesis by activating *mitf* and its downstream key melanogenesis genes such as *tyr*. Therefore, in addition to enhancing melanocyte proliferation, Klf9 may also influence the process of melanogenesis [77]. Furthermore, in the Black-AT-RNA prediction results, the transcription factor Egr1 was also identified. Egr1 is a zinc-finger transcription factor belonging to the EGR family. Studies have shown that EGR1 is an important transcription factor in melanogenesis. α-MSH upregulates the expression of EGR1, which then binds to specific EGR1 binding sequences (EBS) on the *stat3* gene promoter, promoting the transcription of *stat3*. The expression of *stat3* is crucial for α-MSH-induced *tyr* expression [78]. There was no significant difference in the gene expression levels of Klf9 and Egr1 between the black spot and red regions, but numerous studies indicate that Klf9 and Egr1 have a significant impact on melanogenesis. Therefore, we speculate that these two transcription factors may be key factors in the melanogenesis process, but they likely need to cooperate with other transcription factors to synthesize and accumulate melanin at specific sites to form the black spot.

Both Red-AT-RNA and Red-AE-RNA predicted the transcription factor *prdm1*, which is significantly upregulated in the red region (*p* = 2.53 × 10^−6^, log_2_(fc) = −2.4709). Prdm1 belongs to the PRDM family and plays a critical role in the immune system, hematopoietic system, nervous system, and embryonic development [79,80,81]. Studies have found that the upregulation of *prdm1* may enhance the expression of *dkk1* through a non-classical pathway, thereby inhibiting the activity of the Wnt signaling pathway. Dickkopf Wnt signaling pathway inhibitor 1 (DKK1) is a negative regulator of melanocyte differentiation and inhibits melanogenesis and melanocyte differentiation by suppressing the Wnt signaling pathway. Prdm1 indirectly affects the activity of the Wnt signaling pathway by regulating DKK1, thus influencing melanocyte generation and differentiation [82]. Additionally, Red-AT-RNA and Red-AE-RNA also predicted the transcription factor Sp5. This transcription factor is also significantly upregulated in the red region (*p* = 0.0005, log_2_(fc) = −5.3837). Sp5 belongs to the specificity protein/Kruppel-like factor (SP/KLF) family and has a DNA-binding domain composed of a zinc finger structure, which can specifically bind to the GC box in the gene promoter region, regulating the transcription of genes. *Sp5* is one of the downstream target genes of the Wnt signaling pathway and plays a negative feedback role in the Wnt pathway by inhibiting the expression of other Wnt target genes, such as *axin2* and *lef1*, thus regulating the intensity and balance of Wnt signaling. The Lymphoid enhancer-binding Factor 1 (LEF1) plays an important role in melanogenesis, so Sp5 may inhibit melanogenesis [83]. We also found binding sites for Prdm1 and Sp5 on genes that inhibit melanogenesis, such as *cebpa* and *ndrg2*, suggesting that these two transcription factors may influence the expression of these key genes. In conclusion, Prdm1 and Sp5 may inhibit melanogenesis by suppressing the Wnt signaling pathway. Additionally, genes related to the Wnt signaling pathway, such as *wnt3a*, *LOC100710461 (wnt7a)*, and *LOC100690824 (wnt5b)*, are expressed at significantly higher levels in the black spot region than in the red region, indirectly supporting the role of Prdm1 and Sp5 in inhibiting the Wnt signaling pathway in the red region.

In summary, through chromatin state analysis combined with epigenomic and transcriptomic data, we performed motif analysis to identify the motif sequences of key regulatory sites, followed by transcription factor prediction. This led to the identification of several key gene regulatory sites and transcription factors associated with black spot formation. We have preliminarily explored the molecular mechanisms underlying black spot formation in red tilapia, laying the foundation for improving the skin color differentiation of red tilapia. However, given the dynamic nature of gene expression, the functional roles of these candidate genes and transcription factors require further validation through ap-proaches such as gene-editing technologies in future studies.

## 4. Materials and Methods

### 4.1. Experimental Animals and Sample Collection

The methods of all experiments were carried out in accordance with the Guide for the Care and Use of Experimental Animals of China.

**Figure 7:**
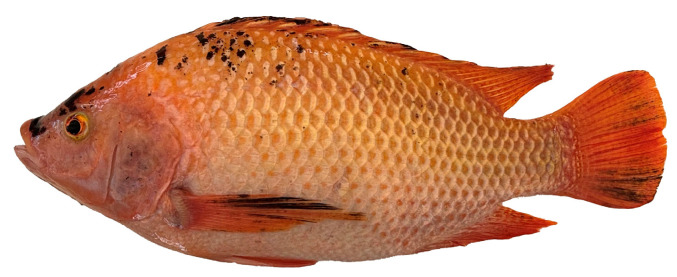
Diagram of red tilapia.

Twelve-month-old red tilapia (average body length 35 ± 1 cm), were obtained from Hainan Baolu Aquatic Science and Technology Co., Ltd. (Lingao County, China). All fish were selected from the same cohort exhibiting black spot pigmentation and were reared under identical outdoor pond conditions. The fish were fed a commercial compound diet (Quanxing brand) and maintained at a density of 10 fish/m^3^. Rearing conditions included a water temperature of 26–30 °C, pH of 7.0–8.0, dissolved oxygen higher than 6 mg/L, and ammonia nitrogen content lower than 0.5 mg/L. Individuals exhibiting prominent black spots on the skin, along with overall good vitality and health, were selected for sampling (Figure 7).

For each selected fish, skin tissue samples (including the dermis) were collected from both the black spot regions and the adjacent red areas. Each fish was treated as a biological replicate, with a total of three fish sampled. The samples from each fish were divided into two parts, one for RNA-Seq and the other for CUT&Tag experiments. Before sampling, the individuals to be sampled were deeply anesthetized with a 0.1–0.2 mg/L eugenol solution and their surfaces were washed with sterile PBS. After sampling, the samples were immediately frozen in liquid nitrogen and then stored in a −80 °C freezer for preservation.

### 4.2. RNA Extraction, Transcriptome Sequencing, Quality Control, and Alignment

Total RNA was extracted from the red tilapia samples using TRIzol (Invitrogen, Carlsbad, CA, USA). RNA integrity was assessed using agarose gel electrophoresis, and RNA purity was measured using a Nanodrop 2000 (Thermo Fisher Scientific, BRIMS, Cambridge, MA, USA). Library construction and sequencing were performed by Beijing Berry Genomics Biotechnology Co., Ltd. The raw sequencing data were first quality-assessed using FastQC (v0.12.0) [84], and then filtered to obtain clean reads using Fastp software (v0.24.1) [85]. The clean reads were aligned to the Nile tilapia reference genome *O_niloticus*_UMD_NMBU (RefSeq assembly accession: GCF_001858045.2) using STAR software (v2.7.11b) [86]. Gene expression counts were calculated using featureCounts [87], and gene expression levels were quantified using the TPM method.

### 4.3. DEGs and Enrichment Analysis

Differential gene expression analysis between the black spot regions and the surrounding red areas in the skin tissue of red tilapia was performed using the DESeq2 software (v1.48.0) on the Omicshare bioinformatics cloud platform, with normalization based on the median-of-ratios method [88]. Genes with *p* < 0.05 and an absolute log₂ fold change (|log₂FC|) > 1 were considered differentially expressed. The GO annotation information database of all genes in the Nile tilapia genome and the KEGG annotation information database were used as background files, and the differential gene set was analyzed by functional enrichment and pathway enrichment using the Omicshare, and the *q* < 0.05 of GO term or map pathway as significantly enriched term or pathway.

### 4.4. Skin Tissue Nucleus Extraction and CUT&Tag Library Construction and Sequencing

NE1 buffer was added to a 1.5 mL centrifuge tube, the tissue block was quickly clipped and poured into Dounce (MERCK, Darmstadt, Germany) to replenish the NE1 buffer, and was ground in a smooth manner using pestle and mortar A and B in turn, and then filtrated through 70 μm and 40 μm cell sieves sequentially to collect the filtrate containing the nuclei. The supernatant was removed by centrifugation, and the precipitate was washed with Wash buffer for 1–2 times, and finally the precipitate was resuspended in Wash buffer and set aside. A portion of the nucleus solution was taken to check the integrity of the nucleus shape using Taipan blue staining, the number of nuclei was determined using a cell counter, and CUT&Tag assay was performed with 50–100,000 nuclei/sample. After that, four histone modifications, H3K4me1, H3K4me3, H3K27me3, H3K27ac, and negative control IgG were used for library construction. Two replicates were set for each histone modification and IgG. To ensure the comparability of data among different samples, *Escherichia coli* λDNA was used as the reference standard for DNA spike-in, and the concentration was set at 5 ng/µL, with a recommended addition rate of 1 pg/100,000 cells. Refer to Vazyme#TD904 Hyperactive Universal CUT&Tag Assay Kit for Illumina Pro operation manual for the steps of library building experiments. Sequencing of the self-constructed libraries was performed by Tianjin Novogene. NE1 buffer, Wash buffer and the antibodies used in the experiments are shown in Table S5.

### 4.5. CUT&Tag Sequencing Data Analysis

The steps of the analysis method in this study refer to Steven Henikoff [89] published in https://www.protocols.io/ (accessed on 13 April 2024) CUT&Tag Data Processing and Analysis Tutorial, on the basis of the source code, combined with their own sequencing data and species characteristics, and adjusted the source code to data analysis was performed.

After assessing the quality of sequencing data using FastQC (v0.12.0) [84], Bowtie2 (v2.5.4) [90] was used to construct the genome index and compare the sequencing data to the genome. Then the files were processed using tools such as samtools (v1.17) [91] and bedtools (v2.28.0) [92] for format conversion. Afterwards, the sequencing data were aligned to the spike-in genes using Bowtie2 software (v2.5.4) to obtain the number of fragments on the alignment, the normalization factor scale_factor was calculated based on the number of fragments obtained, and the data were normalized using the normalization factor, and finally the normalized bedgraph file was obtained. Subsequently, peak identification was performed with Seacr (v1.3) [93] and the bam files were sorted using the samtools software (v1.17), and then the fragment site information was extracted, combined with the GTF file, and the Peak and TSS signal heatmaps were drawn using deeptools software (v2.0) [94]. Finally, the bw files were imported into Integrative Genomics Viewer (IGV) software (v2.18.2) [95], and after constructing the index with the GTF file of the genome, the distribution and intensity of Peak signals on the genome were observed.

Peak signal annotation and visualization were performed using the ChIPseeker (v3.21) [96] and ggplot2 (v3.5.1) [97] packages. An annotation database was constructed based on the species’ genome annotation file. After loading the bedgraph file of the Peak signals, annotation was performed, and a distribution map of the Peak signals across the genomic structural elements was plotted.

Differential analysis of histone modification signals between the black spot and red regions of skin tissue was conducted using the DESeq2 (v1.48.0) package in R (v4.4.1) [98,99], applying a significance threshold of *p* < 0.05 and |log_2_FC| > 1 for differential peaks. Differential Peak regions were identified and annotated using the ChIPseeker software (v3.21). The annotation was based on the nearest gene TSS to the region, thereby associating the differential Peak regions with specific genes.

### 4.6. Chromatin State and Joint Transcriptome Analysis

Chromatin state analysis was performed using ChromHMM software (v1.20) based on the method published by Jason Ernst et al. in 2017 [47]. The four histone modification bam files obtained from the CUT&Tag data analysis were selected as input files, with IgG as the control, and analyzed separately for the black spots and red regions. The BinarizeBam command was used to convert the bam files into binary signal files required by ChromHMM: java -mx4000M -jar ChromHMM.jar BinarizeBam -gzip -f 5 chromsize.txt bamfiles Black_sheet.txt outfiles. After that, the LearnModel command was used to perform automatic classification of chromatin states and enrichment analysis, identifying functional characteristics of different chromatin regions: java -mx4000M -jar ChromHMM.jar LearnModel -color 0,0,255 -p 80 -i chrhmm outfiles 8 rti. Finally, the sites of different chromatin states were annotated to genes, and the relationship between these sites and specific genes was determined.

Based on the results of the chromatin state analysis of the black spots and red regions, the unique active TSS sites or active enhancer sites in the black spots region were selected and intersected with the upregulated genes in the black spots region obtained from the transcriptome data. Similarly, the unique active TSS sites or active enhancer sites in the red region were selected and intersected with the upregulated genes in the red region obtained from the transcriptome data.

### 4.7. Motif and Transcription Factor Prediction

Based on the information from the differential analysis, the sequence fragments of the intersecting genes were extracted and output in fasta format. The MEME Suite [100] official website (https://meme-suite.org/) was accessed on 20 December 2024, and the MEME tool was used for de-novo motif prediction, selecting motifs with a length between 6–20 base pairs, *e* < 0.05, and *p* < 0.05. Afterward, the Tomtom tool on the website was used to predict transcription factors for the motifs that met the criteria.

## 5. Conclusions

In summary, this study integrates multi-omics data to reveal the differences in melanogenesis regulation between the black spot and red regions, clarifying the role of key genes and regulatory elements in black spot formation. We found that the black spot region has a high melanogenic activity, while the red region exhibits inhibition of melanogenesis. This suggests that, under normal conditions, red tilapia may need to suppress melanogenesis to maintain its red color, and the appearance of the black spot may result from the release of this inhibition. Additionally, through histone modification differential analysis, we identified several regulatory elements that may influence black spot deposition. Combined with transcriptomic data, we also identified key transcription factors affecting pigmentation. This study indicates that histone modification, as an epigenetic regulation, plays a crucial role in the formation of black spots in red tilapia. In the future, using higher-resolution techniques such as single-cell CUT&Tag and single-cell RNA-seq could further precisely reveal the specific cell populations where epigenetic regulation occurs, leading to changes in the expression of downstream genes and reactivating melanocytes that were previously suppressed in certain areas of red tilapia skin. The findings of this study not only deepen our understanding of color regulation mechanisms in aquaculture animals but also provide a theoretical foundation for future genetic editing approaches to improve the skin color of red tilapia.

## Figures and Tables

**Table 1:** Statistics of histone modification signals in red tilapia skin samples.

Sample	Black_Peaks	Red_Peaks
H3K4me1	9806	22,822
H3K4me3	2185	2334
H3K27ac	41,748	79,260
H3K27me3	34,812	51,589

Note: Black: black spots region; Red: red region. The number of histone peaks in the table is the average of two biological replicates.

**Table 2:** Statistics of differential histone modification signals in red tilapia skin samples.

Sample	Diff_Peaks	Diff_Genes
H3K4me1	3542	2765
H3K4me3	564	562
H3K27ac	1175	1032
H3K27me3	2274	1952

Note: Diff_Peaks: differential peaks; Diff_Genes: differential genes.

**Table 3:** Key differential peaks in red tilapia skin tissue and their impact on melanin synthesis.

Sample	Gene Name	Gene Annotation	log_2_FC	Function	Reference
H3K4me1	*ctnnb1*	catenin (cadherin-associated protein), beta 1	1.12	promote	Bejaoui M, et al. [34]
H3K4me1	*wnt3*	wingless-type MMTV integration site family, member 3	1.39	promote	Guo H, et al. [35]
H3K4me1	*LOC112846512*	calcium/calmodulin-dependent protein kinase type II delta chain-like	1.49	promote	Hsieh C-C, et al. [36]
H3K4me1	*creb3l2*	cAMP responsive element binding protein 3-like 2	1.31	promote	Jiang K, et al. [37]
H3K4me1	*kitlg*	kit ligand	3.22	promote	Grichnik J M, et al. [38]
H3K4me1	*LOC102082978*	endothelin-3	1.85	promote	Saldana C A, et al. [39]
H3K4me1	*bambi*	BMP and activin membrane-bound inhibitor (Xenopus laevis) homolog	1.92	promote	Marwitz S, et al. [40]
H3K27ac	*kitlg*	kit ligand	2.96	promote	Grichnik J M, et al. [38]
H3K27ac	*kit*	KIT proto-oncogene, receptor tyrosine kinase	1.29	promote	Wang Z Q, et al. [41]
H3K4me1	*nf1*	neurofibromin 1	−5.15	inhibit	Powell M B, et al. [42]
H3K27ac	*foxo6b*	forkhead box O6 b	−2.55	inhibit	Moon K M, et al. [43]
H3K27me3	*LOC100698110*	dickkopf-related protein 1	2.47	inhibit	Yamaguchi Y, et al. [44]
H3K27ac	*tgfbr2l*	transforming growth factor beta receptor-like	−2.93	inhibit	Serre C, et al. [45]
H3K27me3	*LOC100700558*	mothers against decapentaplegic homolog 4	1.27	inhibit	Yang G, et al. [46]

Note: log_2_FC > 0 indicates upregulation in the black spot region; log_2_FC < 0 indicates downregulation in the black spot region. “Promote” refers to the promotion of melanogenesis; “inhibit” refers to the inhibition of melanogenesis.

## Data Availability

The sequencing data for CUT&Tag and RNA-seq generated in this study have been submitted to the NCBI BioProject database (https://www.ncbi.nlm.nih.gov/bioproject/) (accessed on 13 March 2025) under accession number PRJNA1235938 and PRJNA1235842, and it will be made publicly available upon publication.

## Supplementary Materials

The following supporting information can be downloaded at: https://www.mdpi.com/article/10.3390/ijms26094370/s1.
