# Supplementary material for: Integrated Transcriptomic and Epigenomic Analysis Reveals Mechanisms Underlying Melanotic Spot Formation in Red Tilapia (Oreochromis spp.)
Source: Int J Mol Sci. 2025 May 4;26(9):4370. doi: 10.3390/ijms26094370 (PMC12072769; doi:10.3390/ijms26094370)
Supplement: Supplementary file 1 [file ijms-26-04370-s001.zip › Supplementary figures.pdf]

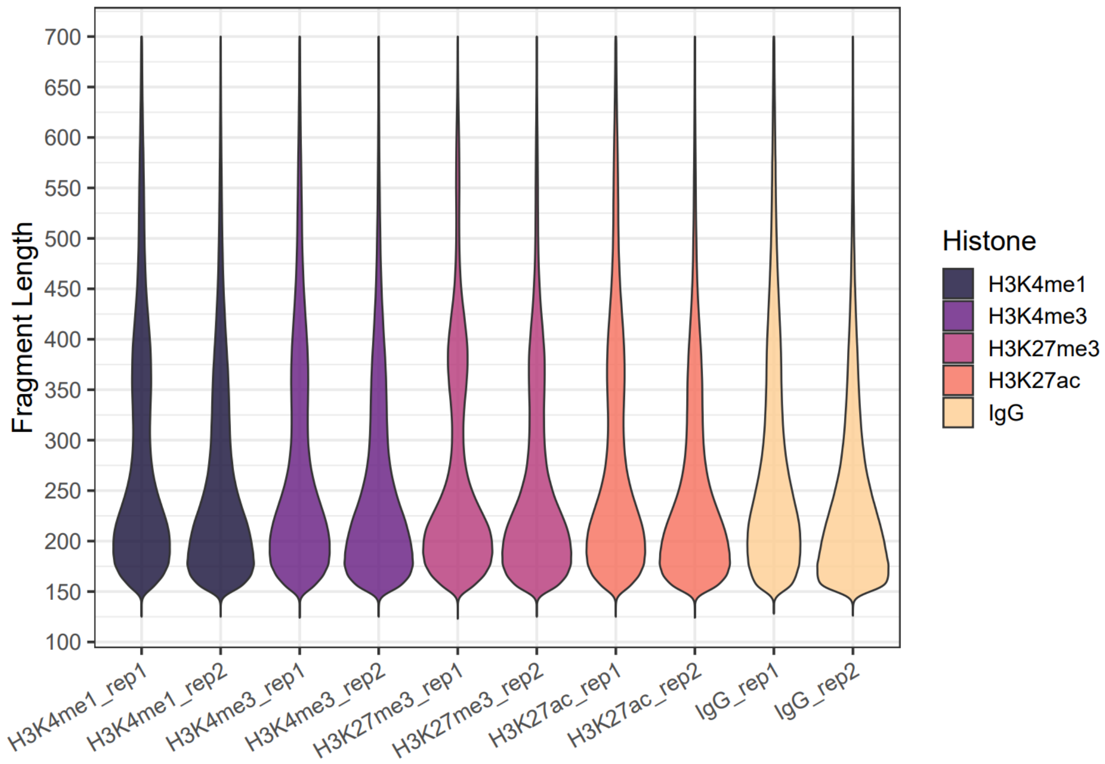

Figure S1. Fragment length distribution of CUT&Tag samples in the red region.

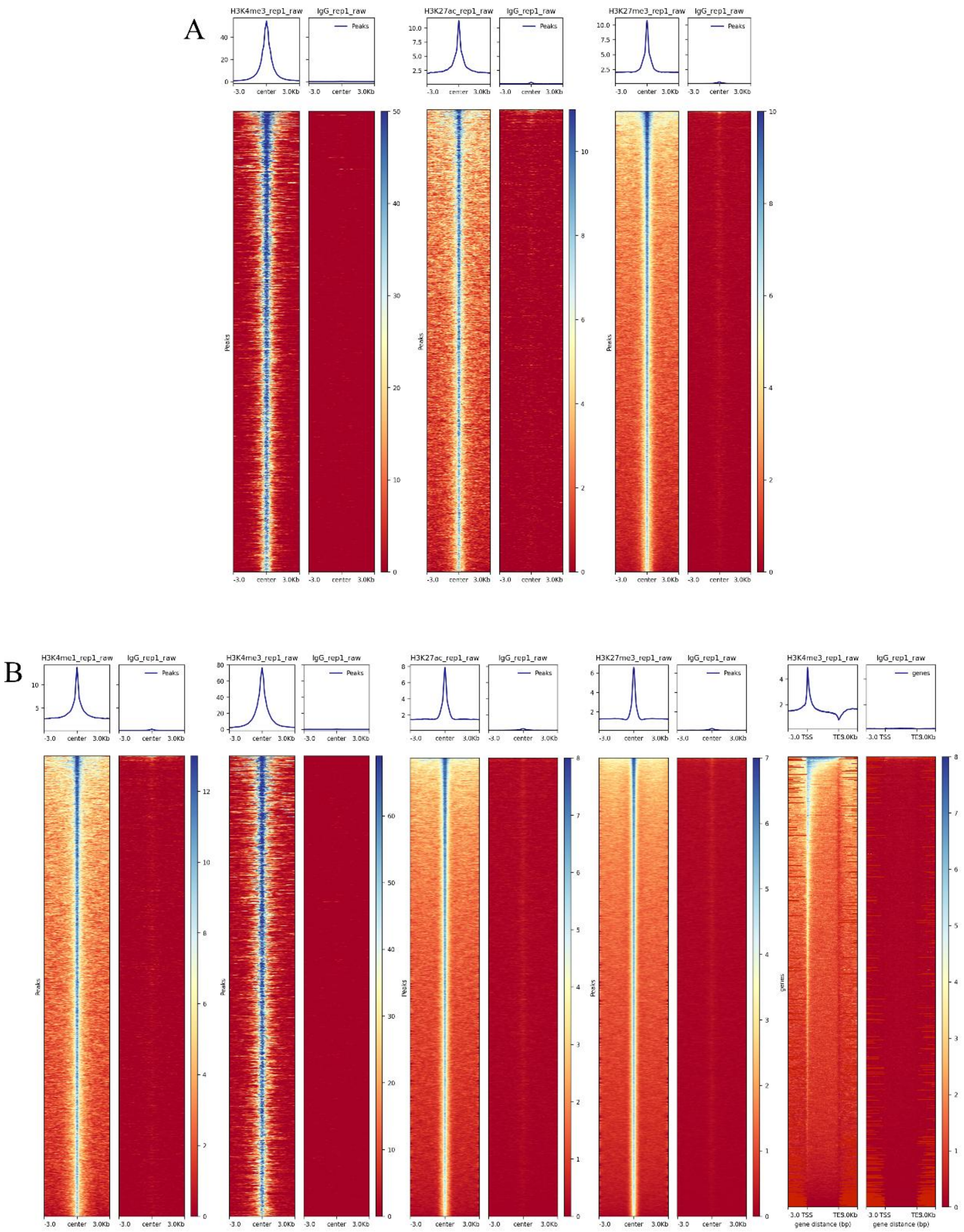

Figure S2. Peak Signal Heatmap. (**A**) Heatmap of peak regions for H3K4me3, H3K27ac, and H3K27me3 in the black spot regions. (**B**) Heatmap of peak regions for four histone modifications in the red regions and the enrichment heatmap of H3K4me3 at the transcription start sites.

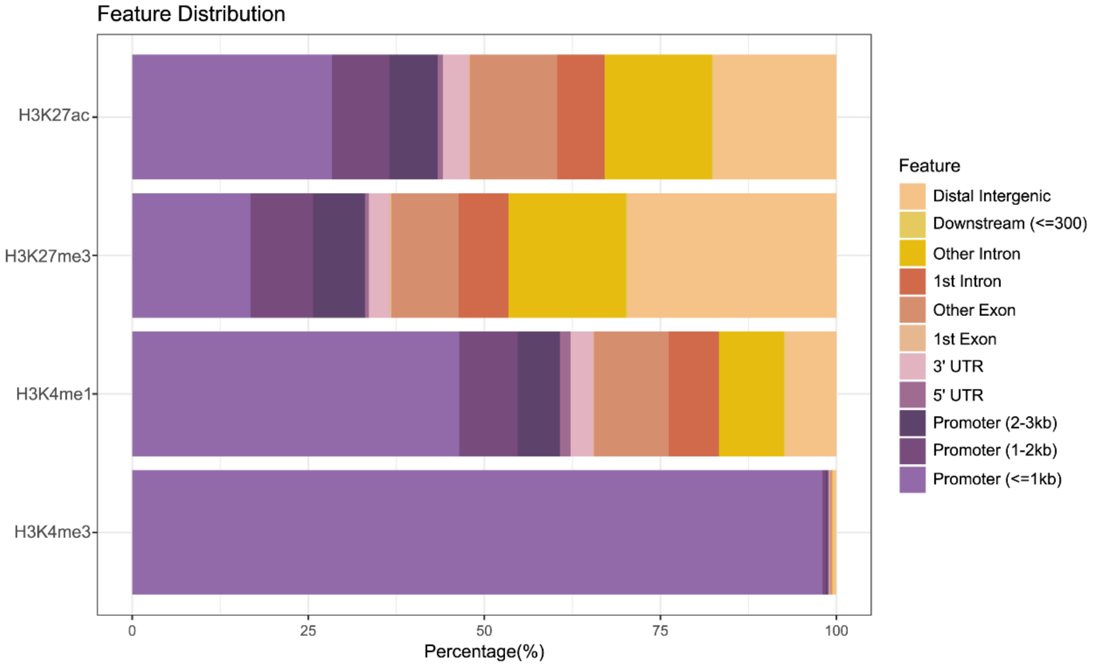

Figure S3. Peak distribution in the functional regions of the red regions.

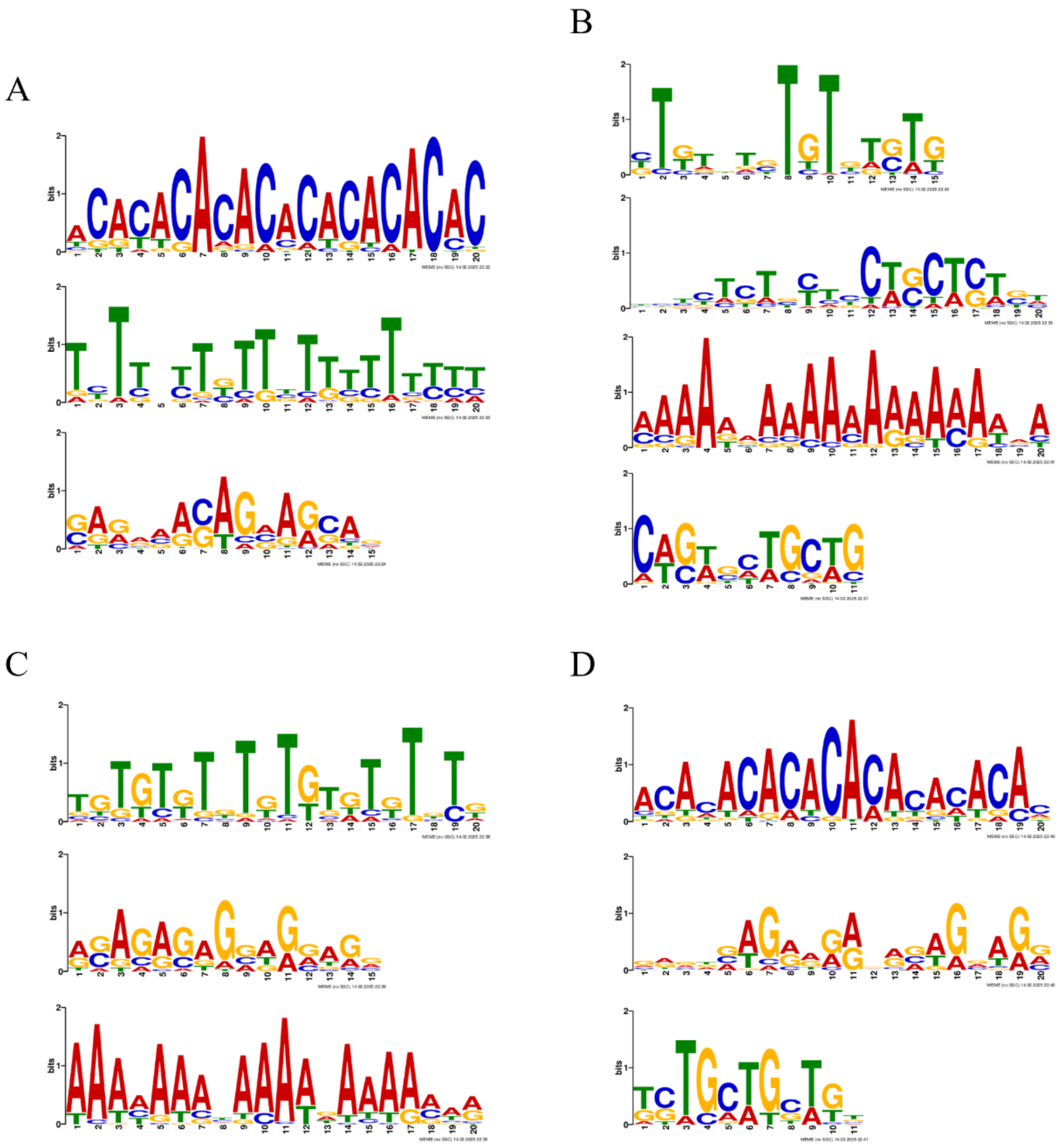

Figure S4. Motif Prediction. (A) Black-AT-RNA motif prediction results, sorted by significance from top to bottom, same below. (B) Black-AE-RNA motif prediction results. (C) Red-AT-RNA motif prediction results. (D) Red-AE-RNA motif prediction results.
