# Supplementary material for: Integrated Transcriptomic and Epigenomic Analysis Reveals Mechanisms Underlying Melanotic Spot Formation in Red Tilapia (Oreochromis spp.)
Source: Int J Mol Sci. 2025 May 4;26(9):4370. doi: 10.3390/ijms26094370 (PMC12072769; doi:10.3390/ijms26094370)
Supplement: Supplementary file 1 [file ijms-26-04370-s001.zip › Supplementary tables.pdf]

**Table S1.** Transcriptome sequencing and quality control results of *Oreochromis* spp.

| Sample | Raw reads | Clean reads | Efficacy/% | Q20/% | Q30/% | GC/% |
|---|---|---|---|---|---|---|
| B_Skin_1 | 24,612,208 | 24,485,293 | 99.48 | 98.03 | 94.58 | 47.12 |
| B_Skin_2 | 28,679,708 | 28,502,810 | 99.38 | 98.08 | 94.72 | 47.90 |
| B_Skin_3 | 33,226,616 | 32,938,096 | 99.13 | 97.87 | 94.27 | 47.82 |
| R_Skin_1 | 27,705,224 | 27,415,654 | 98.95 | 98.25 | 95.12 | 46.55 |
| R_Skin_2 | 21,669,277 | 21,411,993 | 98.81 | 98.24 | 95.00 | 47.37 |
| R_Skin_3 | 22,564,588 | 22,336,361 | 98.99 | 98.23 | 95.00 | 47.00 |

B: black spots region; R: red region. Numbers 1, 2, and 3 represent biological replicates.

**Table S2.** Transcriptome alignment results of skin tissues of *Oreochromis* spp.

| Sample | Total reads | Total mapped | Multiple mapped | Uniquely mapped |
|---|---|---|---|---|
| B_Skin_1 | 24,485,293 | 23,085,853 (94.28%) | 1,229,746 (5.02%) | 21,856,107 (89.26%) |
| B_Skin_2 | 28,502,810 | 26,947,367 (94.54%) | 994,747 (3.49%) | 25,952,620 (91.05%) |
| B_Skin_3 | 32,938,096 | 31,315,958 (95.07%) | 1,108,811 (3.37%) | 30,207,147 (91.71%) |
| R_Skin_1 | 27,415,654 | 26,105,571 (95.22%) | 1,059,401 (3.86%) | 25,046,170 (91.36%) |
| R_Skin_2 | 21,411,993 | 20,477,025 (95.63%) | 712,278 (3.33%) | 19,764,747 (92.31%) |
| R_Skin_3 | 22,336,361 | 21,433,383 (95.95%) | 801,800 (3.59%) | 20,631,583 (92.37%) |

B: black spots region; R: red region. Numbers 1, 2, and 3 represent biological replicates.

**Table S3.** CUT&Tag sequencing and quality control results of *Oreochromis* spp skin tissue.

| Sample | Raw reads | Clean reads | Error/% | Q30/% | GC/% |
|---|---|---|---|---|---|
| B_H3K4me1 | 5,762,863 | 5,056,718 | 0.02 | 92.64 | 44.17 |
| B_H3K4me3 | 3,451,088 | 2,741,401 | 0.01 | 92.94 | 45.70 |
| B_H3K27ac | 7,617,437 | 6,619,270 | 0.02 | 92.35 | 44.29 |
| B_H3K27me3 | 5,111,189 | 4,572,193 | 0.02 | 92.57 | 44.59 |
| B_IgG | 4,190,569 | 3,111,468 | 0.01 | 93.63 | 45.88 |
| R_H3K4me1 | 7,116,055 | 5,181,956 | 0.02 | 91.59 | 44.81 |
| R_H3K4me3 | 7,084,559 | 4,954,063 | 0.02 | 92.06 | 46.06 |
| R_H3K27ac | 8,164,041 | 5,510,619 | 0.02 | 91.93 | 44.72 |
| R_H3K27me3 | 6,102,206 | 4,616,801 | 0.02 | 91.85 | 44.71 |
| R_IgG | 6,557,363 | 3,541,130 | 0.02 | 91.75 | 45.30 |

B: black spots region; R: red region. The data in the table are the average values of histone modification Repeat 1 and Repeat 2.

**Table S4.** CUT&Tag alignment results of *Oreochromis* spp skin tissue.

| Sample | Clean reads | Alignment _Genome/% | Alignment _spike-in/% |
|---|---|---|---|
| B_H3K4me1 | 5056718 | 89.34 | 2.10 |
| B_H3K4me3 | 2741401 | 81.84 | 2.72 |
| B_H3K27ac | 6619270 | 87.92 | 1.33 |
| B_H3K27me3 | 4572193 | 87.61 | 2.22 |
| B_IgG | 3111468 | 61.84 | 10.59 |
| R_H3K4me1 | 5181956 | 92.24 | 0.57 |
| R_H3K4me3 | 4954063 | 88.28 | 5.17 |
| R_H3K27ac | 5510619 | 90.00 | 1.45 |
| R_H3K27me3 | 4616801 | 92.06 | 0.42 |
| R_IgG | 3541130 | 79.95 | 8.42 |

B: black spots region; R: red region. The data in the table are the average values of histone modification Repeat 1 and Repeat 2.

**Table S5.** Information on NE1 buffer, Wash buffer, and Antibody

| | Reagent | Volume | Manufacturer |
|---|---|---|---|
| NE1 Buffer | HEPES-KOH pH=7.9（1mol/L） | 1 mL | Solarbio |
| | Spermidine（2 mol/L） | 12.5 μL | Sigma-Aldrich |
| | KCL（1mol/L） | 500 μL | Sinopharm Chemical Reagent |
| | 10% Triton X-100 | 500 μL | Sinopharm Chemical Reagent |
| | glycerol | 10 mL | Sinopharm Chemical Reagent |
| | ddH2O | Up to 50 mL | — |
| | Roche Complete Protease Inhibitor EDTA-Free | 1 slice | Sigma-Aldrich |
| Wash Buffer | HEPES pH 7.5（1 mol/L） | 1 mL | Solarbio |
| | NaCL（5mol/L） | 1.5 mL | Sinopharm Chemical Reagent |
| | Spermidine（2 mol/L） | 12.5 μL | Sigma-Aldrich |
| | ddH2O | Up to 50 mL | — |
| | Roche Complete Protease Inhibitor EDTA-Free | 1 slice | Sigma-Aldrich |
| Antibody | Anti-Histone H3 (mono methyl K4) antibody | | Abcam |
| | Anti-Histone H3 (tri methyl K4) antibody | | Abcam |
| | Anti-Histone H3 (tri methyl K27) antibody | | Abcam |
| | Anti-Histone H3 (acetyl K27) antibody | | Abcam |
| | Rabbit IgG | | Abcam |
| | Guinea Anti-Rabbit IgG | | Abcam |
